# Circadian Misalignment and Chrononutrition in Type 2 Diabetes: Mechanistic Insights and Translational Gaps

**DOI:** 10.3390/ijms27198473

**Published:** 2026-09-23

**Authors:** İsmail Celil Haskoloğlu, Zeynep Güneş Özünal

**Affiliations:** 1 Department of Pharmacology, Faculty of Pharmacy, Near East University, 99138 Nicosia, Cyprus; ismailcelil.haskologlu@neu.edu.tr; 2 Department of Medical Pharmacology, Faculty of Medicine, Istanbul Atlas University, Kagithane, Anadolu Cad. No: 40, Istanbul 34408, Türkiye

**Keywords:** circadian rhythm, diabetes mellitus, chrononutrition, meal timing, time-restricted eating, circadian misalignment, glucose variability, melatonin, *MTNR1B*, chronopharmacology

## Abstract

Circadian rhythms regulate glucose metabolism, insulin secretion, hepatic glucose production, peripheral insulin sensitivity, nutrient handling, and energy balance. Observational studies have associated circadian timing disruption through shift work, irregular sleep, artificial light at night, social jet lag, late eating, or inconsistent meal timing with insulin resistance and type 2 diabetes mellitus (T2DM). This narrative review integrates molecular, clinical, nutritional, and pharmacological evidence linking circadian misalignment with diabetes, with emphasis on chrononutrition, time-restricted eating, glucose variability, diabetes-related complications, melatonin/*MTNR1B*-related susceptibility, exogenous melatonin, continuous glucose monitoring (CGM), digital phenotyping, and chronopharmacology. A structured narrative literature search was conducted to identify relevant studies on circadian biology, diabetes, meal timing, time-restricted eating, melatonin signaling, *MTNR1B*, circadian biomarkers, CGM, and time-informed therapeutic strategies. Mechanistic and preclinical studies establish clock–metabolism coupling, whereas controlled human physiological studies support time-of-day variation in insulin secretion, postprandial responses, and glucose handling; diabetes-specific interventional and complication-outcome evidence remains limited. Chrononutrition and time-restricted eating may support glycemic regulation only as individualized adjuncts to standard diabetes care, not as replacements for established treatment; medication review and hypoglycemia monitoring are essential, particularly for patients using insulin or insulin secretagogues. Translation into routine care requires longer diabetes-specific trials, standardized outcomes, validated biomarkers, and patient-centered implementation protocols.

## 1. Introduction

Diabetes mellitus represents a major public health challenge because of its rising prevalence, chronic complications, and substantial healthcare burden [1]. Type 2 diabetes mellitus (T2DM), which accounts for most diabetes cases, results from interactions among genetic susceptibility, insulin resistance, pancreatic β-cell dysfunction, adiposity, diet, physical inactivity, sleep behavior, and environmental exposures [2]. Beyond impaired glucose regulation, evidence increasingly implicates the temporal organization of metabolism in glycemic homeostasis and diabetes-related metabolic risk [3].

Circadian rhythms are endogenous, approximately 24 h oscillations that coordinate physiology and behavior with the external light–dark cycle [4]. The suprachiasmatic nucleus synchronizes peripheral clocks in metabolically active tissues, including the pancreas, liver, skeletal muscle, adipose tissue, and gastrointestinal tract [5], while CLOCK–BMAL1 and PER–CRY feedback loops regulate daily variation in metabolic gene expression and hormone signaling [6]. In humans, glucose tolerance, insulin secretion, hepatic glucose output, incretin responses, and peripheral insulin sensitivity vary across the day [7]. Controlled misalignment studies demonstrate adverse effects on glucose tolerance and β-cell compensation [8]; however, real-world associations involving shift work, irregular sleep, artificial light at night, social jet lag, and late eating are also influenced by sleep duration, energy intake, diet quality, physical activity, medication use, and chronotype [9]. Distinguishing causal circadian effects from these co-occurring behavioral factors is therefore essential.

Chrononutrition examines how meal timing, eating regularity, daily energy distribution, and feeding–fasting patterns interact with circadian physiology [10]. Clinical studies have investigated earlier energy intake, avoidance of late-night eating, and time-restricted eating (TRE), but the reported glycemic effects remain heterogeneous [11]. The independent contribution of timing is difficult to separate from changes in caloric intake, body weight, dietary composition, sleep, and behavioral regularity. Differences in eating-window timing, intervention duration, diabetes status, glucose-lowering medication use, and outcome selection further limit comparisons across studies. Evidence obtained in healthy participants, individuals with obesity, or prediabetes also cannot be assumed to apply directly to established T2DM, particularly in patients using insulin or insulin secretagogues. Meal timing may therefore also interact with endogenous melatonin signaling, particularly when food intake overlaps with the biological night.

Melatonin signaling illustrates the context-dependent relationship between circadian biology and glucose regulation [12]. Human crossover evidence indicates that the glycemic response to late eating is influenced by the overlap between food intake and biological night and may differ according to *MTNR1B* genotype [13]. Biological markers such as dim light melatonin onset and cortisol rhythms can characterize internal circadian phase [14], whereas continuous glucose monitoring (CGM) and wearable devices provide time-stamped metabolic or behavioral patterns but do not directly measure endogenous circadian phase. Chronopharmacology likewise provides a biological rationale for time-dependent variation in drug disposition and response [15], but physiological rhythmicity alone does not establish an optimal dosing time for a specific antidiabetic therapy.

Circadian disruption has well-characterized mechanistic and short-term human physiological links with impaired glucose metabolism [16,17], whereas diabetes-specific interventional evidence is less mature and evidence that circadian interventions prevent or modify diabetes-related complications remains preliminary. The unresolved translational question is how far this evidence supports clinical action. Building on prior reviews of circadian mechanisms [3], chrononutrition and TRE [11], and chronotherapeutics [15], the present review integrates these domains within a single evidence-maturity framework and critically evaluates the progression from mechanistic plausibility to diabetes-specific clinical evidence. The review focuses primarily on adults with prediabetes and T2DM; studies in healthy participants or individuals with overweight or obesity are included only when they clarify controlled human physiology, while preclinical studies are used to establish mechanistic plausibility. It distinguishes mechanistic plausibility, preclinical evidence, observational human associations, controlled human physiological evidence, and diabetes-specific interventional evidence; examines why findings concerning meal timing, TRE, melatonin–*MTNR1B* signaling, glucose variability, diabetes-related complications, and chronopharmacology are inconsistent; identifies the methodological bottlenecks limiting causal inference; and distinguishes the resulting implications as currently actionable, conditionally applicable, or research-only. The clinical conclusions are not intended to be extrapolated to type 1 or gestational diabetes.

## 2. Review Framework and Literature Search

This review used a structured narrative approach and targeted literature searches to assess the directness and translational maturity of the available evidence.

PubMed/MEDLINE, Scopus, and Web of Science were searched through June 2026. These searches were supplemented by Google Scholar searches and reference-list screening. Search concepts combined diabetes and glucose regulation with terms related to circadian misalignment, chrononutrition, meal timing, time-restricted eating (TRE), continuous glucose monitoring (CGM), diabetes-related complications, melatonin/*MTNR1B*, circadian biomarkers, wearable technologies, digital phenotyping, and chronopharmacology. A representative search query was: (“circadian misalignment” OR “circadian disruption”) AND (diabetes OR prediabetes).

The review focused primarily on studies involving adults with prediabetes or type 2 diabetes mellitus (T2DM). Evidence from other human populations and preclinical models was included when it provided relevant physiological or mechanistic context but was not used to draw T2DM-specific clinical conclusions.

Primary human studies included randomized and nonrandomized intervention studies, observational studies, and controlled physiological studies. Eligible studies evaluated exposures or interventions related to circadian disruption, meal timing, eating windows, melatonin, or treatment timing and reported glycemic, metabolic, safety, or complication-related outcomes. Studies without a relevant timing exposure or intervention or without a diabetes-related outcome were excluded. Titles and abstracts were screened for relevance, potentially relevant full-text articles were examined, and reference lists were checked to identify additional studies.

Evidence was grouped according to study type and interpreted in light of differences in timing, intervention design, study population, medication use, and outcome selection. Within this review, “actionable” refers to established diabetes-care practices; “conditionally applicable” to adjunctive approaches requiring individualized assessment and appropriate supervision; and “research-only” to strategies lacking sufficient clinical validation for routine implementation. These categories provide a narrative interpretive framework rather than a formal evidence-grading system.

Figure 1 and Figure 2 were conceptually developed by the authors, generated with assistance from ChatGPT (OpenAI; GPT-5.5; accessed in August 2026), and assembled and edited using Microsoft PowerPoint (Microsoft Corporation). All graphical content was reviewed and verified by the authors.

## 3. Circadian Timing in Diabetes: Mechanistic Basis and Clinical Relevance

The circadian system temporally organizes glucose homeostasis by coordinating insulin secretion, hepatic glucose production, peripheral glucose uptake, hormonal signaling, and feeding–fasting behavior. Its relevance to diabetes therefore lies in the timing of metabolic functions rather than merely in the presence of a molecular clock [18]. Core clock components are distinct from clock-associated metabolic regulators. CLOCK, BMAL1, PER, and CRY generate circadian oscillations, whereas REV-ERB, ROR, SIRT1, and AMPK contribute to rhythm stability, energy sensing, and clock–metabolism coupling. Although biologically interconnected, these factors are not established routine biomarkers or therapeutic targets in diabetes [19].

### 3.1. Core Clock Mechanisms Relevant to Metabolic Regulation

At the cellular level, CLOCK and BMAL1 activate PER and CRY transcription, after which PER–CRY complexes inhibit CLOCK–BMAL1 activity. Post-translational processes, including phosphorylation, ubiquitination, degradation, and nuclear transport, regulate the phase and amplitude of this feedback loop [20]. Secondary loops involving REV-ERB and ROR, together with metabolic regulators such as SIRT1 and AMPK, connect circadian transcription to nutrient availability, mitochondrial activity, and cellular energy status [21]. This evidence establishes a mechanistic basis for temporal metabolic regulation but does not demonstrate the clinical efficacy of clock-directed interventions in diabetes.

### 3.2. Central–Peripheral Clock Coordination and Metabolic Timing

The suprachiasmatic nucleus is entrained primarily by retinal light input and coordinates internal rhythms, while peripheral clocks in the pancreas, liver, skeletal muscle, adipose tissue, and gastrointestinal tract also respond to feeding, hormones, autonomic signals, and metabolic state [22]. Human physiological studies demonstrate time-of-day variation in glucose tolerance, insulin sensitivity, postprandial glucose responses, and substrate handling [23]. These findings support the relevance of metabolic timing but do not establish that modifying behavioral or treatment timing improves long-term glycemic or complication-related outcomes.

### 3.3. Tissue Clocks and Diabetes-Relevant Glucose Regulation

Most tissue-specific evidence remains mechanistic or preclinical. Experimental disruption of pancreatic β-cell clocks impairs glucose-stimulated insulin secretion and glucose tolerance [24], while hepatic clock models demonstrate temporal regulation of gluconeogenesis, glycogen metabolism, and related metabolic pathways [25]. Skeletal muscle and adipose clocks have likewise been linked to glucose uptake, lipid flux, inflammatory signaling, and insulin responsiveness, although much of this evidence derives from cellular, animal, or integrative mechanistic studies [26].

Human evidence is narrower. Daily variation in glucagon and incretin responses has been demonstrated in controlled studies involving healthy participants [27], supporting temporal regulation of endocrine metabolism. Extrapolation to T2DM requires caution because diabetes status, medication use, sleep disruption, and altered meal timing may modify these rhythms. Table 1 summarizes the key circadian domains and their clinical implications.

**Table 1:** Circadian domains, diabetes-relevant mechanisms, clinical/metabolic implications, and translational interpretation.

Circadian Domain	Main Diabetes-Relevant Mechanism	Clinical/Metabolic Implication	Translational Interpretation	Reference(s)
Core molecular clock	CLOCK–BMAL1 and PER–CRY feedback loops regulate rhythmic transcription of metabolic genes	Temporal regulation of glucose metabolism, lipid metabolism, mitochondrial function, and energy balance	Provides mechanistic rationale for time-dependent metabolic regulation, but core clock genes are not routine clinical biomarkers in diabetes	[28]
Central–peripheral clock coordination	The SCN synchronizes peripheral clocks through light, autonomic, hormonal, behavioral, and feeding-related cues	Central–peripheral desynchronization may impair coordinated glucose homeostasis	Establishes the mechanistic basis of central–peripheral coordination; direct diabetes-specific clinical application remains unvalidated.	[29]
Pancreatic β-cell and hepatic clocks	β-cell clocks regulate insulin secretion; hepatic clocks regulate gluconeogenesis, glycogen metabolism, and lipid pathways	Postprandial glucose excursions, impaired β-cell responsiveness, fasting hyperglycemia, and altered hepatic glucose output	Strong preclinical mechanistic support, but limited direct human intervention evidence in diabetes.	[30]
Skeletal muscle, adipose, and gut/endocrine rhythms	Muscle clocks affect glucose uptake; adipose clocks regulate lipid flux and inflammation; gut/endocrine rhythms influence incretin and hormonal responses	Peripheral insulin resistance, altered lipid metabolism, glycemic variability, and impaired postprandial regulation	Supports a multi-organ view of circadian metabolic regulation in diabetes	[31]
Circadian misalignment in diabetes care	Misalignment of sleep, light, meal timing, activity, and medication schedules disrupts metabolic timing	Evening hyperglycemia, nocturnal dysglycemia, glucose variability, and medication–meal mismatch	Human studies support acute effects; CGM-guided care remains unvalidated.	[32]

Critical synthesis: Tissue-clock studies provide strong biological plausibility, whereas direct human evidence is strongest for short-term variation in glucose physiology and the acute metabolic effects of circadian misalignment. Evidence that clock-directed interventions improve HbA1c, hypoglycemia, or diabetes-related complications remains limited [33]. Accordingly, the following sections prioritize human evidence on meal timing, time-restricted eating, glucose monitoring, and clinically measurable outcomes while treating longer-term translational claims as hypothesis-generating [34].

## 4. Chrononutrition in Diabetes: Meal Timing, Time-Restricted Eating, Clinical Evidence, and Safety

Chrononutrition examines how the timing, regularity, and daily distribution of food intake interact with metabolic regulation [35]. Feeding–fasting cycles act as entrainment signals for peripheral clocks in metabolically active tissues [36]. Mechanistic pathways, including clock–microbiome interactions, support biological plausibility but do not establish clinical benefit in diabetes [37].

### 4.1. Meal Timing and Glycemic Responses

Postabsorptive and postprandial glucose regulation involves both insulin-dependent and insulin-independent processes [38]. In a randomized crossover study involving participants without diabetes, later meal timing produced less favorable postprandial metabolic responses [39]. In contrast, direct evidence from T2DM remains limited, although one randomized trial found that concentrating more daily energy at breakfast and less at dinner reduced overall daily hyperglycemia [40]. Evidence from gestational diabetes represents a distinct physiological and therapeutic context and is not extrapolated here to prediabetes or T2DM [41].

In adults with dysglycemia, glucose responses following a late eating occasion were associated with overnight and next-morning glucose, although meal composition and insulin sensitivity influenced these relationships [42]. In a small real-world pilot involving impaired glucose tolerance and T2DM, however, meal timing did not significantly affect group-level postprandial glucose responses [43]. These findings indicate substantial inter-individual heterogeneity and do not support a universal clock-time prescription.

Current diabetes guidance favors individualized nutrition care based on metabolic goals, treatment regimen, and patient preferences [44]. Meal-timing changes therefore require coordination with insulin or insulin-secretagogue use [45] and established glucose-lowering therapy [46]. Chronotype may also modify meal-timing associations, but available evidence is largely observational and not diabetes-specific [47].

### 4.2. Time-Restricted Eating: Rationale, Clinical Evidence, and Inconsistencies

Time-restricted eating (TRE) confines daily food intake to a consistent window without necessarily prescribing caloric restriction [48]. Proposed mechanisms include reduced late-night intake, a longer overnight fast, and stronger feeding–fasting signals for peripheral clocks [49]. However, trials conducted mainly in adults with overweight or obesity have produced mixed results [50,51], demonstrating that timing effects are difficult to separate from spontaneous energy restriction, weight change, and adherence.

Reviews and meta-analyses report possible improvements in body weight and selected glycemic or cardiometabolic outcomes [52,53,54]. Nevertheless, findings vary according to population, eating-window timing and duration, comparator, medication use, and intervention length [55]. Comparisons of early, late, and self-selected windows have also not demonstrated consistent metabolic differences [53]. In diabetes trials, CGM can characterize 24 h glucose patterns and hypoglycemia, but these outcomes must complement HbA1c, adverse-event reporting, and medication assessment rather than substitute for them [56].

Critical synthesis: As summarized in Table 2, current evidence does not support a uniform or exclusively circadian effect of TRE. Benefits vary by population and outcome, frequently coexist with lower energy intake or weight loss, and have also been observed with later eating windows, while effects on insulin sensitivity remain inconsistent [57,58,59,60,61,62,63]. The independent contribution of meal timing may also be difficult to separate from changes in dietary composition, including macronutrient distribution, glycemic index, and fiber content, representing an additional translational gap. TRE is therefore best regarded as a feasible adjunct for selected patients rather than a proven timing-specific therapy independent of energy balance [64].

### 4.3. Safety, Patient Selection, and Clinical Boundaries

Small controlled studies and circadian reviews support a physiological rationale for earlier eating [65,66], and controlled human evidence indicates impaired evening meal tolerance in T2DM [67]. Nevertheless, direct long-term comparisons of early and late TRE in diabetes remain insufficient to establish clinical superiority, while energy balance remains a major competing explanation for benefit [68].

TRE differs from intermittent fasting regimens that alter intake across whole days [69,70,71]. Neither approach can be considered a circadian intervention unless eating-window timing is measured. Similarly, benefits accompanied by reduced energy intake or weight loss cannot be attributed to timing alone.

Medication safety determines eligibility. Patients using insulin or sulfonylureas require medication review and glucose monitoring because fasting responses may differ according to therapy [72]. Recurrent hypoglycemia, unstable glycemic control, frailty, pregnancy, eating-disorder history, advanced kidney disease, or dehydration risk require specialist assessment rather than unsupervised implementation [45,64,72]. Frailty may further complicate medication adherence and glycemic management [73]. TRE also does not inherently improve diet quality, so concurrent nutritional support may remain necessary [74]. Supervised TRE may be considered for selected adults with stable prediabetes or T2DM as an adjunct to, not a replacement for, standard diabetes care; no validated responder phenotype has been established [63,69].

## 5. Circadian Misalignment, Glucose Variability, and Diabetes-Related Complications

Circadian misalignment refers to a mismatch between endogenous circadian timing and behavioral or environmental cycles such as sleep, light exposure, food intake, physical activity, and work schedules. In diabetes, the clinical relevance of circadian misalignment extends beyond disease risk alone [75]. It may also be associated with daily glycemic patterns, postprandial glucose excursions, nocturnal dysglycemia, and glucose variability, whereas evidence that it independently influences the progression of diabetes-related complications remains limited. Therefore, circadian disruption should be considered not only as a potential contributor to T2DM development but also as a factor that may shape metabolic control after diabetes has been established [76].

Lifestyle and environmental drivers of circadian misalignment often coexist. Shift work may combine nighttime light exposure, sleep restriction, irregular eating, altered physical activity, and psychosocial stress [77]. Similarly, social jet lag, evening screen exposure, late meals, and inconsistent sleep timing may interact to weaken the coordination between central and peripheral clocks. These combined exposures can disturb hormonal rhythms, desynchronize tissue clocks, alter metabolic gene expression, and impair glucose regulation [78].

### 5.1. Shift Work, Artificial Light, Sleep Disruption, and Behavioral Desynchronization

Shift work is a prominent real-world model of chronic circadian disruption because night or rotating schedules misalign endogenous rhythms with sleep, activity, and food intake. Epidemiological studies associate long-term shift work with obesity, insulin resistance, metabolic syndrome, and T2DM, whereas controlled human studies indicate that short-term circadian misalignment can impair glucose tolerance and insulin sensitivity and produce inadequate β-cell compensation [79].

Artificial light at night, including evening screen exposure, may suppress melatonin secretion and shift circadian phase. Experimental bright-light exposure during the biological night has acutely altered glucose and insulin responses [80]. These findings provide direct human evidence of short-term metabolic effects; their long-term relevance to glycemic control and diabetes-related complications remains to be established.

Sleep duration, quality, and regularity are also linked to metabolic health. Sleep restriction may impair insulin sensitivity, alter appetite-regulating hormones, increase sympathetic activity, and disrupt glucose-regulatory rhythms [81]. Social jetlag has been associated with obesity, insulin resistance, and metabolic syndrome. However, shift work, nocturnal light, sleep disruption, late eating, physical inactivity, and psychosocial stress frequently coexist, making their independent circadian effects difficult to isolate [82]. In established diabetes, these exposures are most clinically relevant when they disrupt coordination among sleep, meals, medication, physical activity, and glucose monitoring.

### 5.2. Glucose Variability, Nocturnal Dysglycemia, and CGM Patterns

A key diabetes-specific research question is whether circadian disruption contributes to glucose variability. Glycemic control is often assessed by HbA1c, but HbA1c does not capture the timing, amplitude, or distribution of glucose excursions across the day and night [83]. Circadian misalignment may influence glycemic patterns by shifting food intake into periods of lower glucose tolerance, altering hepatic glucose output, impairing insulin sensitivity, and changing sleep-related hormonal regulation. These mechanisms may contribute to higher postprandial excursions, nocturnal hyperglycemia, or unstable overnight glucose profiles [84].

Continuous glucose monitoring (CGM) offers a useful tool for evaluating circadian–metabolic patterns in diabetes. CGM can identify time-of-day variation in glucose excursions, nocturnal dysglycemia, morning hyperglycemia, time in range, time above range, and hypoglycemia frequency [85]. These metrics may be particularly relevant for chrononutrition and TRE interventions, because the clinical goal is often not only to reduce average glucose, but also to improve the temporal distribution of glucose exposure. CGM-derived outcomes can complement HbA1c and fasting glucose by characterizing temporal glucose patterns, but CGM does not directly measure endogenous circadian phase [86].

Circadian misalignment may also interact with medication timing and hypoglycemia risk. For example, patients using insulin or insulin secretagogues may experience different glycemic risks depending on the timing of meals, fasting intervals, sleep schedules, and physical activity. In this context, circadian disruption is not simply a background lifestyle factor; it may directly influence the safety and interpretation of glucose-lowering therapy [87].

### 5.3. Cardiovascular Risk, Blood Pressure Rhythms, and Autonomic Regulation

Cardiovascular disease remains one of the major causes of morbidity and mortality in diabetes. Circadian regulation is relevant to cardiovascular risk because blood pressure, heart rate, vascular tone, endothelial function, coagulation, sympathetic activity, and inflammatory signaling show daily variation [88]. In a controlled crossover study involving healthy adults, acute circadian misalignment increased blood pressure and selected inflammatory risk markers [89]. These acute effects provide direct human experimental support for the cardiovascular relevance of circadian misalignment, while their long-term clinical implications in diabetes require disease-specific evaluation.

Shift work and irregular sleep timing may contribute to cardiometabolic risk through repeated disruption of autonomic and endocrine rhythms. In patients with diabetes, these effects may be clinically important because hypertension, dyslipidemia, inflammation, and glycemic variability often coexist [90]. Although causal pathways remain difficult to isolate, circadian disruption may represent an additional layer of risk that interacts with established cardiovascular risk factors rather than replacing them [91].

### 5.4. Circadian Disruption and Microvascular Complications

Microvascular complications are driven by chronic hyperglycemia, blood pressure, inflammation, oxidative stress, and endothelial dysfunction. Circadian regulation of renal, retinal, and neuronal functions adds a potential temporal dimension [92].

Renal circadian rhythms influence blood pressure, sodium handling, and hemodynamics [93]. In T2DM, evening candesartan reduced morning albuminuria more than morning dosing, supporting dosing-time relevance alongside established renal management [94,95].

Retinal circadian and melatonin-related pathways provide mechanistic support [96]. Evening chronotype was associated with higher retinopathy incidence or progression independently of mean HbA1c [97], complementing established screening and management [98].

Neuropathy may intersect with circadian biology through sleep disruption, pain rhythms, autonomic dysfunction, and inflammation [99,100]. Adjunctive bedtime melatonin improved pain and sleep interference in painful diabetic neuropathy [101], supporting short-term symptom management rather than disease modification. Table 3 summarizes the number and types of evidence sources on circadian factors and diabetes-related complications.

**Table 3:** Number and type of evidence sources represented in this review for circadian factors and diabetes-related complications.

Complication	Primary Human Evidence Represented	Review/Mechanistic Evidence	Clinical Guidance	Interpretation
Cardiovascular disease	One controlled crossover study in healthy adults [89]; no diabetes-specific outcome study	Two reviews [88,91]	One diabetes guideline [90]	Findings in healthy adults provide physiological context but not evidence of cardiovascular benefit in diabetes.
Diabetic nephropathy	One randomized antihypertensive dosing-time study in adults with T2DM and albuminuria [94]	Two mechanistic/review sources [92,93]	One diabetes guideline [95]	Evening candesartan produced a greater reduction in morning albuminuria than morning dosing, providing direct timing-specific human evidence.
Diabetic retinopathy	One prospective cohort study in 731 adults with T2DM assessing chronotype and retinopathy incidence/progression [97]	Two mechanistic/review sources [92,96]	One diabetes guideline [98]	Evening chronotype was associated with higher retinopathy incidence/progression after adjustment for mean HbA1c, supporting diabetes-specific clinical relevance.
Diabetic neuropathy	One randomized, double-blind, placebo-controlled adjunctive bedtime melatonin trial in 103 adults with T2DM and painful diabetic neuropathy [101]	Three mechanistic/review sources [92,99,100]	One diabetes guideline [98]	Adjunctive bedtime melatonin improved pain and pain-related sleep interference, providing direct short-term human intervention evidence for symptom management.

Counts reflect the evidence sources identified through the structured narrative search described in Section 2 and characterize the balance between primary, review-based, and guideline evidence. Evidence from healthy or non-diabetic participants is presented only as physiological context and should not be interpreted as direct evidence for the prevention or treatment of diabetic complications.

### 5.5. Clinical Interpretation

The strongest evidence linking circadian disruption with diabetes relates to metabolic risk, glucose tolerance, insulin sensitivity, sleep disruption, shift work, and meal timing. Evidence for direct effects on diabetes-related complications is less mature, but biologically plausible [102]. The main clinical value of this framework is that circadian misalignment may help explain why some patients show high evening glucose excursions, nocturnal dysglycemia, variable CGM profiles, poor sleep, or difficulty aligning medication and meal schedules [103].

Circadian misalignment is best understood as a modifiable biological and behavioral dimension that may interact with hyperglycemia, insulin resistance, blood pressure, inflammation, sleep disruption, medication timing, and lifestyle patterns, rather than as an isolated cause of diabetes complications. A key research priority is to determine whether circadian-informed interventions can improve CGM-derived glucose variability, nocturnal dysglycemia, cardiovascular risk markers, and complication-related outcomes in diabetes [104].

## 6. Melatonin, *MTNR1B*, and Exogenous Melatonin in Diabetes-Related Glucose Regulation

Melatonin is a major hormonal output of the circadian system and provides an endocrine signal of biological night. It is primarily secreted by the pineal gland under the control of the suprachiasmatic nucleus and is strongly suppressed by light exposure during the night. Although melatonin is most widely recognized for its role in sleep–wake regulation, it also interacts with metabolic pathways involved in glucose homeostasis, pancreatic β-cell function, insulin secretion, oxidative stress, and energy balance [105].

In the context of diabetes, melatonin is important not simply as a sleep-related hormone but as a temporal signal that may influence the metabolic response to food intake. During the biological night, elevated melatonin levels may contribute to reduced insulin secretion and lower glucose tolerance, consistent with the physiological expectation that food intake is usually minimized during the rest phase [106]. This relationship becomes clinically relevant when carbohydrate-rich meals are consumed late in the evening or close to bedtime, when endogenous melatonin concentrations may already be elevated [107].

Melatonin signaling is therefore best interpreted within a time-dependent framework. The same hormone may support circadian organization and antioxidant defense under some conditions while also limiting insulin secretion when food intake occurs during the biological night [108]. This dual role is particularly important for chrononutrition, because it suggests that the metabolic effects of meal timing may depend not only on clock time but also on the individual’s internal circadian phase, melatonin profile, and genetic susceptibility [109].

### 6.1. Melatonin as a Circadian and Metabolic Signal

Melatonin is synthesized mainly from tryptophan through serotonin-dependent enzymatic pathways, with arylalkylamine N-acetyltransferase and hydroxyindole-O-methyltransferase contributing to its production. However, for diabetes-related metabolic regulation, the most relevant feature of melatonin is not the biochemical pathway of synthesis itself, but its rhythmic secretion pattern and its role as a marker of biological night [110].

The onset, duration, and amplitude of nocturnal melatonin secretion provide information about circadian phase. Dim light melatonin onset is widely used in circadian research as a marker of internal biological timing [111]. In metabolic studies, this is important because the same meal may produce different glycemic responses depending on whether it is consumed during a circadian phase characterized by low or high melatonin signaling [112].

Melatonin also influences metabolic homeostasis through antioxidant, anti-inflammatory, and mitochondrial effects. Clinical syntheses report improvements in inflammatory and oxidative stress markers [113] and a modest reduction in HbA1c, but no significant effect on fasting glucose has yet been demonstrated, with substantial heterogeneity across T2DM trials [114]. These mixed findings illustrate the complexity of clinical translation. Melatonin is neither uniformly beneficial nor harmful in diabetes; its effects appear to depend on timing, dose, receptor signaling, metabolic state, and genetic background, and further research is required to clarify these relationships [115].

### 6.2. Melatonin Receptors and Pancreatic β-Cell Function

The physiological actions of melatonin are mediated primarily through the G protein-coupled receptors MT1 and MT2, encoded by *MTNR1A* and *MTNR1B*, respectively. These receptors are expressed in central circadian structures as well as in peripheral tissues relevant to metabolism. Importantly, melatonin receptors are also expressed in pancreatic islets, supporting a direct link between melatonin signaling and β-cell function [116].

In pancreatic β-cells, melatonin receptor activation can modulate intracellular signaling pathways involved in insulin secretion, including cyclic AMP, calcium signaling, and kinase-mediated pathways [117]. Experimental evidence suggests that melatonin may reduce glucose-stimulated insulin secretion, particularly during the biological night. From a physiological perspective, this may help align insulin release with fasting–feeding cycles. However, when food intake occurs late in the evening, this same mechanism may contribute to impaired postprandial glucose tolerance [118].

Melatonin may also exert cytoprotective effects in β-cells through antioxidant and anti-inflammatory mechanisms. These effects may be relevant under conditions of oxidative stress, glucotoxicity, lipotoxicity, or β-cell injury [119]. However, most protective effects are supported primarily by experimental or preclinical evidence, whereas the inhibitory effects on insulin secretion have more direct relevance to human meal timing and glucose tolerance. For this reason, diabetes-focused interpretation requires a distinction between mechanistic β-cell protection and clinically observable effects on insulin secretion and glycemic responses [120].

### 6.3. MTNR1B Variants and Diabetes Risk

Genetic variation in *MTNR1B* provides one of the strongest links between melatonin signaling and diabetes susceptibility. The rs10830963 variant, particularly the G risk allele, has been associated with higher fasting glucose, impaired early insulin secretion, altered β-cell function, and increased risk of type 2 diabetes in genetic and metabolic studies [121].

Mechanistically, *MTNR1B* risk variants may increase melatonin receptor signaling in pancreatic β-cells, thereby enhancing the inhibitory effect of melatonin on insulin secretion [122]. This provides a plausible biological explanation for why some individuals may show greater impairment in glucose tolerance when eating during periods of elevated melatonin. In this context, *MTNR1B* is not merely a genetic risk marker, but a potential example of how circadian biology, meal timing, and β-cell function may interact [123].

Human studies examining dinner timing and melatonin exposure suggest that late food intake may worsen glucose tolerance, especially when endogenous melatonin levels are high and particularly among carriers of *MTNR1B* risk alleles [124]. These findings are highly relevant to chrononutrition because they support the idea that late eating may be metabolically unfavorable not only because of behavioral habits or caloric excess, but also because of circadian–hormonal interactions [125].

However, *MTNR1B* findings require cautious interpretation. Genetic risk does not determine diabetes development on its own, and the effect of *MTNR1B* variants may interact with age, adiposity, sleep timing, dietary habits, chronotype, medication use, and other metabolic risk factors. Therefore, *MTNR1B* represents a potential contributor to individualized circadian susceptibility rather than a stand-alone diagnostic or therapeutic marker [126].

### 6.4. Exogenous Melatonin: Timing, Dose, Glucose Tolerance, and Safety in Diabetes

Exogenous melatonin requires separate consideration from endogenous melatonin signaling. Endogenous melatonin reflects biological night and contributes to circadian phase organization, whereas exogenous melatonin introduces an additional time-dependent signal that may shift circadian timing, influence sleep, and interact with glucose regulation. Accordingly, the metabolic effects of melatonin supplementation require interpretation in relation to administration time, dose, baseline circadian phase, sleep status, glucose tolerance, and *MTNR1B* genotype [127].

From a chronopharmacological perspective, melatonin is particularly relevant because the same compound may have different metabolic consequences depending on when it is administered [128]. Melatonin taken at a time that improves circadian alignment or sleep regularity may indirectly support metabolic regulation in selected individuals with circadian disruption. However, melatonin exposure that overlaps with food intake during the biological night may impair glucose tolerance or reduce insulin secretion in susceptible individuals, especially when carbohydrate-rich meals are consumed close to bedtime [129].

Current evidence does not support the use of melatonin as a general glucose-lowering therapy for diabetes. Its potential benefits may be more relevant to circadian rhythm sleep–wake disorders, delayed circadian phase, shift work-related misalignment, or sleep disruption than to direct metabolic treatment [130]. In patients with diabetes or impaired glucose regulation, melatonin use requires individualization, with administration timing considered alongside meal timing, medication use, CGM patterns, sleep timing, and the risk of nocturnal dysglycemia [131].

### 6.5. Clinical Interpretation for Personalized Chrononutrition and Time-Informed Care

Individuals with evening chronotype, late dinner habits, delayed sleep timing, or high exposure to artificial light at night may experience greater overlap between food intake and endogenous melatonin secretion. In such individuals, avoiding large carbohydrate-rich meals close to bedtime may be particularly relevant [132].

Melatonin biology provides a physiological rationale for individualized meal timing, including limiting large late-evening meals and considering chronotype [133]. CGM may help identify whether late meals produce exaggerated postprandial glucose excursions in specific patients, while sleep and meal timing records may help clarify whether these responses are related to circadian misalignment [134].

These mechanisms strengthen the rationale for personalized chrononutrition and time-informed metabolic care [135]. Nevertheless, associations between *MTNR1B* genotype and diabetes risk do not establish the clinical utility of genotype-guided meal timing or treatment selection [136]. *MTNR1B* genotyping, melatonin profiling, and melatonin-based chronotherapy remain investigational for personalizing diabetes care and require validation in long-term, well-controlled human studies.

The metabolic relevance of melatonin depends on biological timing, exogenous administration, receptor signaling, genetic background, and the temporal relationship between melatonin exposure and food intake [137]. The most clinically useful interpretation is not that melatonin is a diabetes therapy, but that melatonin biology may help identify when late eating, circadian misalignment, or poorly timed supplementation could worsen glycemic regulation in susceptible individuals [138].

## 7. Clinical Translation: Biomarkers, Digital Phenotyping, Chronopharmacology, and Implementation

Translating circadian biology into diabetes care requires reliable assessment of circadian–metabolic alignment in real-world settings. Clock time alone is insufficient because individuals differ in chronotype, sleep timing, light exposure, work schedules, meal timing, medication use, and endogenous circadian phase [139]. In diabetes, the clinically relevant question is not only whether circadian disruption is present but whether it is associated with postprandial glucose excursions, nocturnal dysglycemia, glucose variability, hypoglycemia risk, or poor alignment between meals, sleep, and medication timing [140].

### 7.1. Measuring Circadian–Metabolic Alignment: Biomarkers, CGM, and Wearables

Dim light melatonin onset (DLMO) is one of the most established markers of internal circadian phase, whereas cortisol rhythms provide information about hypothalamic–pituitary–adrenal axis timing and stress-related endocrine regulation. In metabolic research, these markers can help determine whether food intake, sleep, light exposure, or medication administration occurs during the biological day or biological night [141].

Molecular biomarkers, including rhythmic expression of clock genes such as *CLOCK*, *BMAL1*, *PER*, and *CRY* in peripheral blood mononuclear cells or other accessible tissues, may provide additional information about circadian system function. However, molecular rhythm assessment requires repeated sampling, standardized timing, and careful control of sleep, light exposure, meals, and activity; therefore, it remains primarily a research tool rather than a routine clinical test for diabetes [142]. Metabolomic profiling may also reveal circadian regulation of glucose, lipids, amino acids, bile acids, and energy-related metabolites, but methodological complexity and the need for standardized sampling currently limit its clinical application [143,144].

Continuous glucose monitoring (CGM) provides a clinically relevant metabolic readout for evaluating time-dependent glucose patterns in diabetes [145]. Unlike HbA1c, CGM captures the timing, frequency, and magnitude of postprandial excursions, nocturnal hyperglycemia, time in range, time above range, time below range, and hypoglycemia episodes [146]. These data are especially useful for assessing whether late meals, nocturnal snacking, short sleep, irregular meal timing, or compressed eating windows are associated with disproportionate glycemic responses [147].

However, CGM does not directly measure endogenous circadian phase, and its use to prescribe meal timing specifically for circadian alignment remains investigational. CGM-derived glucose patterns are therefore most informative when interpreted together with sleep timing, meal timing, medication timing, chronotype, light exposure, and, when available, biological circadian markers such as DLMO [148,149]. In chrononutrition and TRE studies, CGM-derived outcomes such as evening glycemic burden, nocturnal dysglycemia, time-of-day glucose excursions, and hypoglycemia frequency may be more informative than fasting glucose alone, particularly in patients using insulin or insulin secretagogues [150].

Behavioral markers such as sleep timing, sleep regularity, rest–activity rhythms, light exposure, meal timing, and eating-window duration provide practical information about circadian alignment because they directly influence glucose patterns, medication schedules, physical activity, and postprandial responses [151]. Actigraphy and wearable technologies can characterize rest–activity rhythms, sleep timing, heart rate, skin temperature, and, in some devices, light exposure in real-world conditions [152]. Nevertheless, wearable-based monitoring requires cautious interpretation because many consumer devices use proprietary algorithms and generally measure behavioral proxies rather than the endogenous circadian clock itself; these tools therefore complement rather than replace gold-standard circadian markers such as DLMO [153,154].

### 7.2. Digital Phenotyping and Artificial Intelligence-Assisted Stratification

Artificial intelligence (AI) and machine-learning approaches may help analyze multimodal datasets that include wearable signals, sleep–wake timing, light exposure, meal timing, CGM data, medication use, chronotype, and clinical characteristics. These methods could support digital circadian phenotyping by identifying patterns of behavioral regularity, circadian disruption, glycemic variability, or response to time-based interventions [155].

At present, wearable-based and AI-assisted circadian phenotyping remain investigational for guiding personalized meal or medication timing in diabetes [156]. Important limitations include limited ground-truth circadian phase measurements, variability among wearable devices, short validation periods, small or non-representative datasets, algorithmic bias, data privacy concerns, and lack of standardized clinical endpoints [157].

In diabetes, AI-assisted models may eventually help identify patients who are more likely to benefit from chrononutrition, earlier meal timing, TRE, sleep regularization, or medication schedule review [158]. However, their use as stand-alone tools for prescribing meal timing, medication timing, or chronotherapy depends on prospective validation in diabetes-specific populations [159]. Overall, DLMO and cortisol rhythms provide information about internal circadian phase, CGM captures clinically meaningful glucose patterns without directly measuring the circadian clock, and wearables characterize behavioral rhythmicity as proxy measures [160]. The most promising near-term use is the integration of sleep, light, activity, meal timing, medication timing, and glucose rhythms to support individualized lifestyle counseling and clinical research rather than automated precision chronotherapy [161].

### 7.3. Chronopharmacology and Circadian-Targeted Therapeutic Opportunities

Chronopharmacology examines how biological timing influences drug disposition, target-tissue sensitivity, and therapeutic response [162]. Current syntheses position chronopharmacology as an emerging translational framework, while evidence supporting routine circadian rescheduling of antidiabetic treatment remains limited [163]. Daily variation in gastrointestinal and renal physiology, hepatic metabolism, insulin sensitivity, β-cell responsiveness, and glucose handling provides a biological rationale for time-dependent pharmacotherapy [164,165]. Demonstrating clinically meaningful benefit requires evaluation using glycemic, safety, adherence, and feasibility outcomes [166].

#### 7.3.1. Current Clinical Medication-Schedule Management

Medication timing involves two distinct questions: when pharmacotherapy is initiated and when individual doses are administered. Earlier antidiabetic medication initiation has been associated with lower all-cause mortality, whereas its association with major adverse cardiovascular events was not statistically significant [167]. This differs conceptually from determining whether a particular clock time improves drug efficacy or safety.

Within-day scheduling is drug-specific because antidiabetic therapies differ in pharmacokinetics, meal association, tolerability, renal considerations, and hypoglycemia risk [168]. Insulin and insulin secretagogues require coordination with food intake, fasting, activity, sleep, and glucose monitoring to limit postprandial hyperglycemia and hypoglycemia [45,150,169,170]. For therapies with lower intrinsic hypoglycemia risk, including SGLT2 inhibitors and GLP-1 receptor agonists, timing remains guided primarily by drug- and formulation-specific labeling, tolerability, meal association, adherence, and individual clinical needs [171,172]. Current evidence does not establish a routine circadian dosing strategy for these agents. Thus, currently actionable medication-timing management concerns schedule coordination and safety rather than a generalized circadian dosing algorithm.

#### 7.3.2. Emerging Dosing-Time Hypotheses and Clinical Validation Needs

Human pharmacokinetic modelling using data from 191 healthy adults indicates that metformin exposure varies by time of day, with modelled variation in glomerular filtration, renal plasma flow, and OCT2 activity as contributing mechanisms [173]. These findings support investigation of metformin dosing time but do not establish a clinically preferred schedule. Melatonin-based approaches remain context-dependent and investigational [174,175,176].

#### 7.3.3. Experimental Clock-Targeted Pharmacology

Complementary systems modelling suggests that metformin may also interact with hepatic circadian networks [177]. REV-ERB, ROR, SIRT1, and AMPK represent additional candidate pathways linking the clock with energy sensing, mitochondrial function, and metabolic transcription [178]. However, these clock-directed approaches remain predominantly mechanistic or preclinical and require diabetes-specific clinical validation.

The strongest near-term contribution of chronopharmacology is the coordination of medication schedules with meals, fasting, sleep, activity, hypoglycemia risk, and glucose patterns. Associations between sleep–meal timing and CGM-derived outcomes in adults with obesity without diabetes support the physiological relevance of temporal coordination but do not establish a drug-timing benefit in diabetes [179]. Current implementation should therefore prioritize medication safety and individualized schedule review, while precision chronotherapy remains an important translational objective.

Future precision strategies may combine CGM, chronotype, sleep monitoring, medication profiles, and circadian biomarkers [180,181]. Their implementation requires drug-specific diabetes trials and integration with established treatment guidance [166,182].

### 7.4. Patient-Centered Implementation and Future Research Priorities

Clinical translation of circadian biology into diabetes care requires a stepwise, patient-centered framework rather than a universal circadian intervention for all patients. A practical starting point is to identify individuals in whom sleep timing, meal timing, work schedules, evening hyperglycemia, nocturnal dysglycemia, medication timing, hypoglycemia risk, or glycemic variability may be clinically relevant [183].

This proposed framework comprises four steps: assessment, stratification, intervention, and monitoring. It combines established diabetes-care practices with investigational timing-based approaches and has not yet been validated as an integrated clinical decision-making algorithm. Assessment includes chronotype, sleep–wake timing, shift-work exposure, light exposure, meal timing, eating-window duration, physical activity timing, medication schedules, hypoglycemia risk, and CGM-derived glucose patterns [184]. Stratification identifies patients with late eating, high evening glucose excursions, nocturnal hyperglycemia, recurrent hypoglycemia, irregular sleep schedules, or meal–medication mismatch. Because evening hyperphagia and nocturnal ingestion may arise from different behavioral backgrounds, similar clock-time patterns do not necessarily indicate the same intervention need [185]. Interventions include regularizing sleep–wake timing, reducing late-night eating, shifting caloric intake earlier when feasible, supervised TRE in selected patients, and reviewing medication timing when meal timing or fasting patterns change [186]. Monitoring includes CGM trends, nocturnal dysglycemia, hypoglycemia, adherence, sleep–meal timing, tolerability, and patient feasibility. The intervention is reassessed when hypoglycemia increases, feasibility deteriorates, or no clinically meaningful benefit is observed.

Within this framework, chrononutrition is a conditionally applicable adjunct to established diabetes care. Earlier meal timing, regular eating schedules, avoidance of large late-evening meals, reduction in nocturnal snacking, and supervised TRE may be considered in selected patients, particularly when CGM shows evening hyperglycemia or high glycemic variability. These strategies complement established diabetes nutrition therapy and require individualization according to insulin or sulfonylurea use, hypoglycemia risk, chronotype, shift work, cultural eating patterns, occupational demands, adherence, and patient feasibility [186].

Digital tools can support implementation by characterizing behavioral and metabolic rhythms in real-world settings. Actigraphy, wearable technologies, meal-timing records, sleep diaries, and CGM measure complementary behavioral or metabolic proxies rather than endogenous circadian phase. AI-assisted digital phenotyping offers further opportunities for risk stratification and intervention design, while its transition to routine clinical decision support depends on prospective validation in diabetes-specific populations [187].

Chronopharmacology and circadian-targeted therapeutics offer important translational opportunities, but implementation depends on drug-specific evidence. Although drug absorption, metabolism, clearance, and tissue responsiveness may vary across the circadian cycle, current evidence is insufficient to support broad changes to antidiabetic medication timing solely on circadian grounds. Medication timing therefore continues to follow drug labeling, meal association, safety considerations, hypoglycemia risk, tolerability, renal function, and clinician guidance unless drug-specific chronopharmacological evidence is available [188].

Several evidence gaps remain. Many chrononutrition and TRE studies are short-term, include small samples, or combine meal-timing changes with caloric restriction, making it difficult to isolate the independent contribution of circadian alignment [189]. Much of the diabetes-specific evidence also remains observational; shift-work studies, for example, establish associations with T2DM risk rather than the efficacy of circadian intervention [190]. Trials remain particularly limited among insulin-treated patients, older adults, shift workers, individuals with multimorbidity, and patients at high risk of hypoglycemia. As summarized in Section 5, direct evidence linking circadian interventions with nephropathy, retinopathy, neuropathy, or cardiovascular outcomes remains preliminary.

Priority research areas include longer randomized controlled trials, standardized circadian and metabolic outcomes, careful reporting of medication use, and stratification by chronotype, sleep timing, shift-work status, meal-timing pattern, *MTNR1B* genotype, and baseline glycemic patterns. An ongoing randomized trial of sleep-timing modification in prediabetes and T2DM illustrates the type of diabetes-specific design required [191]. Relevant glycemic outcomes extend beyond HbA1c and fasting glucose to CGM-derived time in range and hypoglycemia frequency [192]. Nocturnal dysglycemia, postprandial excursions, adherence, tolerability, and safety also warrant systematic assessment. Associations between chrononutrition patterns and multidimensional sleep health further support combined assessment of these behavioral domains [193]. Such studies can determine which patients benefit most from chrononutrition, which eating windows are optimal, whether and how exogenous melatonin can be used or avoided, and how time-based strategies can be safely integrated into personalized diabetes care.

Emerging non-laboratory AI-based risk frameworks [194] and observational associations among chrononutrition, sleep behaviors, and glycemic outcomes in prediabetes [195] further illustrate the potential of integrated behavioral and metabolic phenotyping. Circadian-informed diabetes care is most clinically useful as a practical layer added to established diabetes management.

## 8. Conclusions

Circadian rhythms regulate insulin secretion, hepatic glucose production, peripheral insulin sensitivity, nutrient handling, and energy balance. The evidence reviewed supports the view that diabetes pathophysiology and management are influenced not only by impaired glucose regulation but also by the temporal organization of metabolism. Misalignment involving sleep, light exposure, food intake, activity, or medication timing may disrupt glucose homeostasis. The available clinical evidence is most robust for metabolic and glycemic outcomes; by contrast, evidence that interventions targeting circadian timing can prevent or alter diabetes-related complications remains limited. Against this background, the principal contribution of this review is an evidence-maturity framework that separates mechanistic and physiological observations from diabetes-specific clinical findings and classifies their implications as actionable, conditionally applicable, or research-only.

Within this framework, chrononutrition may complement established diabetes care when appropriately individualized, but it should not replace standard treatment. Beyond chrononutrition, glucose variability, nocturnal dysglycemia, melatonin/*MTNR1B*-related susceptibility, digital phenotyping, and chronopharmacology extend the translational scope of circadian-informed diabetes care.

Future studies require long-term randomized designs with standardized circadian and metabolic measures, CGM-derived endpoints, and safety reporting. Priorities include separating meal-timing effects from energy balance, clarifying how melatonin/*MTNR1B* signaling and circadian disruption influence glucose variability and diabetes-related complications, and identifying patients most likely to benefit from circadian-informed interventions. At present, cautious coordination of behavioral and nutritional timing with medication review and glucose monitoring represents the most defensible clinical approach, while precision chronotherapy remains investigational.

## Figures and Tables

**Figure 1:**
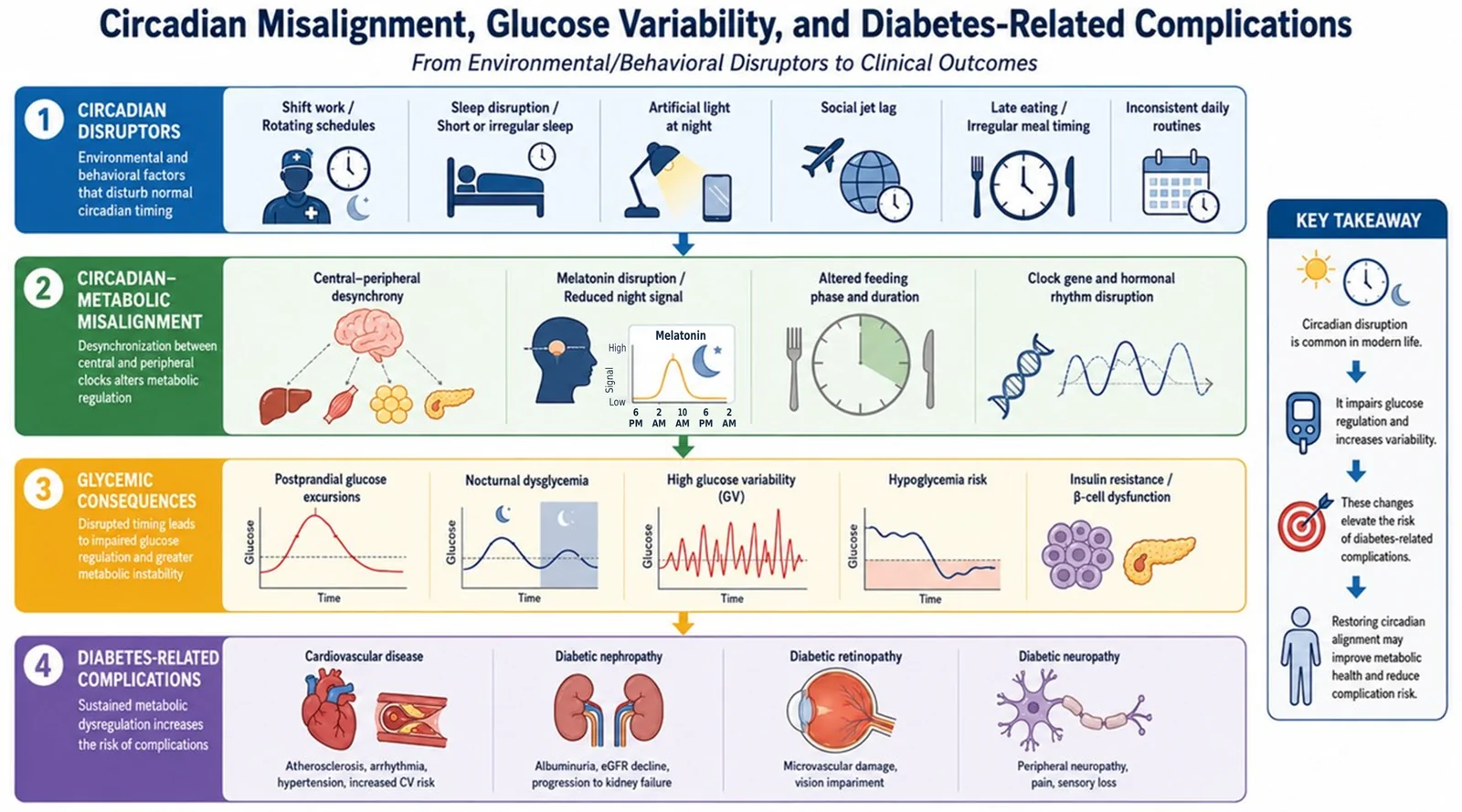
Circadian misalignment, glycemic dysregulation, and diabetes-related complications. The figure was conceptualized by the authors and generated with assistance from ChatGPT (OpenAI; GPT-5.5; accessed in August 2026). The figure was assembled and edited using Microsoft PowerPoint (Microsoft Corporation). All graphical content was reviewed and verified by the authors. No previously published figures or figure elements were reproduced or adapted.

**Figure 2:**
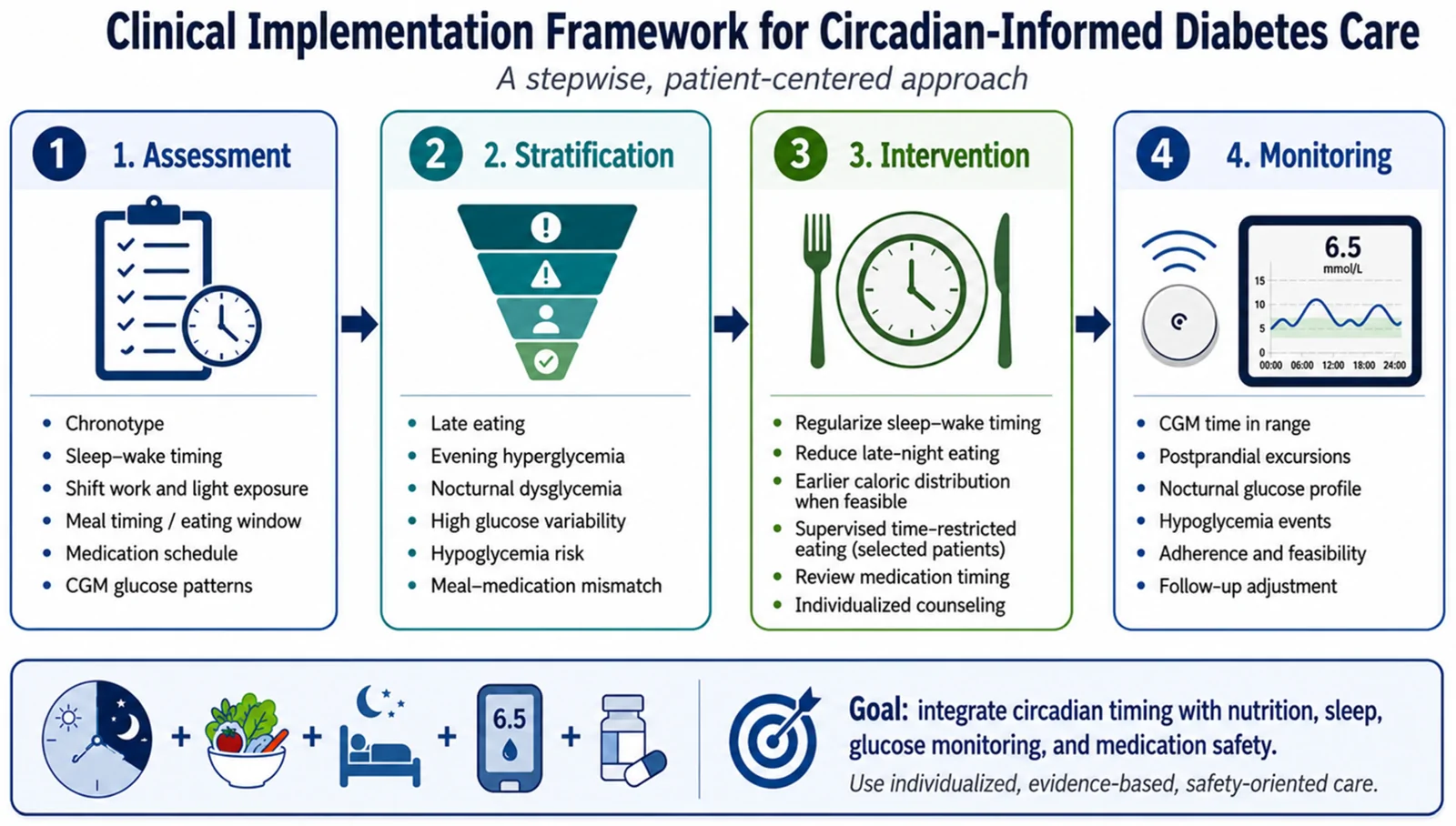
Proposed clinical implementation framework for circadian-informed diabetes care. The figure was conceptualized by the authors and generated with assistance from ChatGPT (OpenAI; GPT-5.5; accessed in August 2026). The figure was assembled and edited using Microsoft PowerPoint (Microsoft Corporation). All graphical content was reviewed and verified by the authors. No previously published figures or figure elements were reproduced or adapted.

**Table 2:** Selected human evidence on time-restricted eating in prediabetes and type 2 diabetes.

Study/Evidence Type	Population and Protocol	Duration/Comparator	Main Findings	Clinical Interpretation and Safety Considerations	References
Systematic review and meta-analysis	Mixed populations including individuals with overweight, obesity, prediabetes, metabolic risk, and some diabetes-related cohorts; TRE protocols varied across studies	Varied; compared with usual diet, control diet, or other dietary interventions	Reported potential improvements in glycemic markers, body weight, and selected cardiometabolic outcomes, but effects varied by protocol, population, and study design	Potential benefit, but marked protocol and population heterogeneity; safety reporting was inconsistent.	[57]
Early time-restricted eating trial	Men with prediabetes; early 6 h eating window with food intake completed earlier in the day	5 weeks; compared with a 12 h control eating window	Improved insulin sensitivity and β-cell responsiveness without requiring weight loss; also improved blood pressure, oxidative stress, and appetite-related outcomes	Weight-independent physiological benefit in prediabetes; the small male cohort limits T2DM generalizability.	[58]
Feasibility study in T2DM	Adults with T2DM, generally diet or oral medication-controlled; 9 h TRE window from 10:00 to 19:00	4 weeks; pre–post habitual eating comparison	TRE was feasible, but no significant changes in body mass or measures of glycemic control were observed.	Feasible in selected patients with T2DM; no significant glycemic benefit was demonstrated.	[59]
Randomized controlled trial in overweight T2DM	Overweight adults with T2DM; 10 h feeding window from 08:00 to 18:00	12 weeks; compared with usual eating pattern/control	HbA1c, body weight, and HOMA-IR improved relative to control; lipid and medication-effect scores also improved	Glycemic benefit, but energy reduction, weight change, and structured follow-up limit attribution to timing alone.	[60]
Randomized crossover trial in T2DM	Adults with T2DM; 10 h TRE window	3 weeks; compared with eating spread over ≥14 h	Improved 24 h glucose homeostasis, increased time in normal glucose range, and reduced time in high glucose range; insulin sensitivity did not improve	Better 24 h glycemia without improved insulin sensitivity, indicating endpoint-specific benefit.	[61]
Randomized clinical trial in T2DM and obesity	Adults with T2DM and obesity; 8 h TRE window from 12:00 to 20:00 without calorie counting	6 months; compared with daily calorie restriction and control	HbA1c decreased similarly with TRE and calorie restriction; only TRE produced significant weight loss relative to control.	Later TRE reduced HbA1c similarly to calorie restriction; accompanying energy reduction and weight loss limit attribution to circadian alignment.	[62]
Systematic review and meta-analysis in T2DM and impaired fasting glucose	Adults with T2DM or impaired fasting glucose; TRE protocols and eating windows varied across included studies.	Varied; intervention durations, protocols, and comparator conditions differed across studies	Modest improvements in fasting glucose, HbA1c, and time in range were reported, but the evidence base was small and heterogeneous.	Modest pooled benefit, but small samples and heterogeneous protocols preclude a universal prescription.	[63]

TRE, time-restricted eating; T2DM, type 2 diabetes mellitus; HbA1c, glycated hemoglobin A1c; HOMA-IR, homeostatic model assessment of insulin resistance. The table presents selected representative studies rather than a comprehensive systematic review.

## Data Availability

No new data were created or analyzed in this study. Data sharing is not applicable to this article.

## Abbreviations

AI	artificial intelligence;
AMPK	AMP-activated protein kinase;
BMAL1	brain and muscle ARNT-like 1;
CGM	continuous glucose monitoring;
CLOCK	circadian locomotor output cycles kaput;
CRY	cryptochrome;
DLMO	dim light melatonin onset;
GLP-1	glucagon-like peptide-1;
HbA1c	glycated hemoglobin A1c;
HOMA-IR	homeostatic model assessment of insulin resistance;
MT1	melatonin receptor 1;
MT2	melatonin receptor 2;
*MTNR1A*	melatonin receptor 1A gene;
*MTNR1B*	melatonin receptor 1B gene;
OCT2	organic cation transporter 2;
PER	period;
REV-ERB	reverse erythroblastosis virus-ErbA-related receptor;
ROR	retinoic acid receptor-related orphan receptor;
SCN	suprachiasmatic nucleus;
SGLT2	sodium-glucose cotransporter 2;
SIRT1	sirtuin 1;
T2DM	type 2 diabetes mellitus;
TRE	time-restricted eating.
